# Dietary Riboflavin Requirement of Pacific White Shrimp (*Litopenaeus vannamei*)

**DOI:** 10.1155/2023/6685592

**Published:** 2023-04-25

**Authors:** Kokila Sanjeewani, Kyeong-Jun Lee

**Affiliations:** ^1^Department of Marine Life Sciences, Jeju National University, Jeju 63243, Republic of Korea; ^2^Marine Science Institute, Jeju National University, Jeju 63333, Republic of Korea

## Abstract

This study was conducted to determine the dietary riboflavin requirement and its effects on growth performance, feed utilization, innate immunity, and diet digestibility of *Litopenaeus vannamei*. A riboflavin-free basal diet (R0) was formulated as a control, and six other diets were prepared by adding riboflavin of 10, 20, 30, 40, 50, and 60 mg/kg to the basal diet (designated as R10, R20, R30, R40, R50, and R60, respectively). Quadruplicate groups of shrimp (initial average weight 0.17 ± 0.00 g) were fed the diets six times a day for 8 weeks. Weight gain, specific growth rate, and protein efficiency ratio were significantly increased by riboflavin (*p* < 0.05). The maximum values were observed in shrimp fed R40 diet. The highest activities of phenoloxidase, nitro blue tetrazolium, superoxide dismutase, and glutathione peroxidase were observed in shrimp fed R40 diet. Lysozyme activity was significantly higher in shrimp fed R30 and R40 diets than that of shrimp fed R60 diet (*p* < 0.05). Intestinal villi were significantly longer in shrimp fed R50 and R60 diets compared to those of all other groups while the shortest villi were observed in R0 group (*p* < 0.05). Intestinal villi were clearly distinguished in shrimp fed higher levels of riboflavin compared to those of shrimp fed R0 and R10 diets. Apparent digestibility coefficients of dry matter and protein in diets were not significantly affected by riboflavin levels (*p* < 0.05). Whole-body proximate composition and hemolymph biochemical parameters were not significantly altered by dietary riboflavin (*p* < 0.05). Therefore, the results of this study indicate that riboflavin is essential to enhance growth performance, feed utilization, nonspecific immunity, and intestine morphology of shrimp. An optimal riboflavin requirement for the maximum growth of *L. vannamei* seems to be approximately 40.9 mg/kg diet.

## 1. Introduction

Riboflavin (vitamin B2) is a water-soluble vitamin synthesized by plants and most microorganisms. It is essential for biochemical reactions in all living cells [1]. It functions as a precursor of two coenzyme forms, flavin adenine dinucleotide (FAD) and flavin mononucleotide (FMN), required for redox reactions and energy production in animals through the metabolism of carbohydrates, lipids, proteins, and ketone bodies [1, 2]. Riboflavin also protects human and animal tissues from oxidative stress by preventing lipid peroxidation and reducing reperfusion oxidative injuries occurred by free radicals and inflammatory cytokines [3]. Riboflavin promotes the conversion of tryptophan into niacin (vitamin B3) together with pyridoxine (vitamin B6) [4] and activates vitamin K, pyridoxine, and folic acid (vitamin B9) to their physiologically active forms [5, 6].

Riboflavin and other vitamins are often produced by gut microbiota in animals [7, 8]. The quantity of vitamins produced via microbial synthesis is generally low in shrimp and other decapods because of their simple digestive tracts compared with mammals and birds [9]. Therefore, the overall riboflavin levels in practical feeds are prone to be insufficient to meet the dietary requirements of decapods despite the presence of riboflavin in many feed materials [10]. The positive effects of dietary riboflavin supplementation have been reported on growth performance, antioxidant capacity, intestinal health, and immune status of aquatic animals [11–18]. An optimum level of dietary riboflavin was also reported to upregulate mRNA expression of tight junction proteins in grass carp, *Ctenopharyngodon idella* [12, 13], and prevent deficiency signs such as anorexia, short-body dwarfism, and cataracts in sunshine bass, *Morone chrysops × M. saxatilis* [14], and channel catfish, *Ictalurus punctatus* [15]. Kumar [16] reported that the optimum level of dietary riboflavin reduced adverse effects of stress given by arsenic pollution (2.68 mg/L) and high temperature (34°C) in striped catfish, *Pangasianodon hypophthalmus*. An optimum level of riboflavin was able to increase intestinal enzyme activities and lipid utilization in Jian carp, *Cyprinus carpio* var. Jian [17], and positively affected RNA/DNA ratio in spotted snakehead, *Channa punctatus* [18]. In shrimp, optimum riboflavin levels in diets for black tiger shrimp, *Penaeus monodon*, and kuruma shrimp, *Penaeus japonicus*, were estimated as 22.3 and 80 mg/kg, respectively [19, 20]. Whole-body riboflavin level of *P. monodon* was increased with the increase of dietary riboflavin levels although the growth, feed utilization, and survival were not significantly affected [19]. However, *P. monodon* showed deficiency signs such as irritability, short-head dwarfism, protuberant cuticle, and light coloration when fed a riboflavin-deficient diet (0.48 mg/kg) [19]. A riboflavin-deficient diet led to decreased growth performance of *P. monodon* and freshwater prawn, *Macrobrachium rosenbergii* [21, 22]. However, Reddy et al. [23] did not observe any adverse effect in *P. monodon* fed riboflavin-deficient diet.

Pacific white shrimp, *Litopenaeus vannamei*, is the most commercially important farmed shrimp species in the world [24]. It is necessary to provide nutritionally balanced feed which meets their nutrient requirements for optimum growth performance, feed utilization efficiency, and health condition. Nutritionally balanced feeds are important to reduce production cost and water pollution in aquaculture industry [25, 26]. The dietary requirements of other vitamins were previously determined for *L. vannamei*, including thiamin (vitamin B1) [27], niacin [28, 29], pyridoxine [30, 31], and biotin (vitamin B7) [32]. However, the optimum riboflavin requirement for *L. vannamei* remains unknown although it is available for *P. japonicus* and *P. monodon*. According to previous studies, low or high dietary vitamin levels lead to reduced shrimp growth and health status [27–30]. Therefore, the present study was designed to determine the dietary riboflavin requirement of *L. vannamei* through its effects on growth performance, feed utilization, innate immunity, antioxidant capacity, diet digestibility, and intestinal histology.

## 2. Materials and Methods

### 2.1. Experimental Diets

A riboflavin-free basal diet (R0) was formulated as a control to contain 100 g/kg fish meal (tuna meal and sardine meal) and 400 g/kg soybean meal as major protein sources. Fish oil was used as a major lipid source. To prepare six additional test diets, riboflavin was added to the control diet at concentrations of 10, 20, 30, 40, 50, and 60 mg/kg (designated as R10, R20, R30, R40, R50, and R60, respectively). Riboflavin-free vitamin mixture was prepared before feed preparation. Ingredients were thoroughly mixed, and a wet dough was prepared by adding lipid source and distilled water (10%). Then, the homogenous mixture was pelleted by using a pelletizer (SP-50, Gum Gang Engineering, Korea) with a 2 mm die. The wet pellets were dried using an electrical dryer at 24-25°C for 8 h to reduce the moisture content to lower than 10%. Then, the dried pellets were stored at –20°C in airtight polyethylene bags until used. The ingredients and proximate composition of the diets are shown in Table 1. The high-performance liquid chromatography (HPLC) method of Hasselmann et al. [33] was used to determine the riboflavin concentration of the diets and found to contain 1.41 ± 0.45 (R0), 6.38 ± 0.76 (R10), 17.87 ± 1.20 (R20), 25.26 ± 0.12 (R30), 37.11 ± 2.21 (R40), 46.01 ± 0.62 (R50), and 55.11 ± 2.63 (R60) mg/kg in the dry diet.

### 2.2. Experiment Setting and Feeding Trial

The experimental shrimp were acclimated for two weeks before the feeding trial. During the acclimatization period, commercial feed (40% protein, 6% lipid Woosung premium aqua feed, Korea) was supplied. Healthy and equal-sized shrimps (0.17 ± 0.00 g) were randomly chosen and transferred to 215 L capacity 28 acrylic tanks. A total of 25 shrimp were stocked into each tank to be one of four replicates per dietary treatment. Filtered and preheated seawater was supplied to each tank and aerated continuously. Feeds were delivered manually six times a day (08 : 00, 10 : 00, 12 : 00, 14 : 00, 16 : 00, and 18 : 00 h) for 8 weeks. Daily feeding rates were 12% of shrimp body weight at the beginning of the study and then gradually decreased to 3% at the end. The bulk weight and total shrimp count of each tank were measured fortnightly to adjust the feed amount according to the growth rate. Approximately 70% of culture water was exchanged with filtered and preheated seawater every 3rd day. Water quality was manipulated and recorded daily. During the feeding trial, different physiochemical parameters such as temperature, pH, dissolved oxygen, and ammonia were routinely recorded as 28.0-31.0°C, 7.47-7.81, 5.70-6.77 mg/L, and 0.04-0.10 mg/L, respectively. Experimental protocols followed the guidelines of the Animal Care and Use Committee of Jeju National University.

### 2.3. Sample Collection

Shrimp were starved for a day before samplings. Individual shrimp in each tank were counted and weighed. The collected data was used to calculate weight gain (WG), specific growth rate (SGR), feed conversion ratio (FCR), protein efficiency ratio (PER), and survival (Table 2). For hemolymph sampling, three shrimp were randomly chosen from each tank (12 shrimp per treatment) and anesthetized by dipping them into an ice water bath. Hemolymph was sampled from the base of the 3rd walking leg puncture with 1 mL syringes with a 25-gauge needle and immediately mixed with the same volume of anticoagulant, Alsever's solution (A3551, Sigma-Aldrich, St. Louis, MO, USA). Hemolymph was centrifuged (800 × *g* for 20 minutes at 4°C) to separate the serum. The supernatant was removed and kept at –80°C for the analysis of the immune and blood parameters. The same shrimp were dissected after collecting hemolymph and approximately 1 cm length midgut samples were collected into Davidson's fixative solution for histological analysis. Three additional shrimp per tank were kept at –20°C to examine the whole-body proximate composition.

### 2.4. Sample Analyses

Moisture and ash content of diets and whole-body were estimated by gravimetric analysis according to standard techniques [34]. Moisture content was measured by drying 2 g of samples in a heated oven at 125°C until a constant weight is obtained. Ash content was determined by burning 2 g of samples in a muffle furnace at 550°C for four hours. According to Folch et al. [35], crude lipid was quantified gravimetrically after extraction with a chloroform-methanol mixture. Crude protein levels were analyzed by the Kjeldahl method (Kjeltec™ 2300, FOSS Analytical, Hilleroed, Denmark) after acid hydrolysis. Intracellular production of the superoxide anion in hemocytes was determined according to nitro blue tetrazolium (NBT) reduction to formazan by hemocytes according to Song and Hsieh [36] with slight modifications [37]. Phenoloxidase (PO) activity of hemocytes was measured by the formation of dopachrome produced from L-dihydroxyphenylalanine (L-DOPA) [38]. Lysozyme activity was measured using a turbidimetric method through the reduction of bacteria (*Micrococcus lysodeikticus*) due to enzyme activity during incubation [39]. According to the manufacturer's instructions, glutathione peroxidase (GPx) activity was measured by using GPx commercial kit (Biovision, Inc. California, USA), superoxide dismutase (SOD) activity was measured by using a SOD commercial kit (Biovision, Inc. California, USA), and total antioxidant capacity (TAC) of shrimp muscle was determined using a commercial kit (CS0790, Sigma-Aldrich, USA). Hemolymph antiprotease activity was measured by determining the percentage inhibition of trypsin activity according to Ellis [40] with slight modifications as applied by Magnadóttir et al. [41]. Levels of cholesterol, glucose, triglyceride, and total protein in hemolymph were analyzed using an automated blood analyzer (SLIM, SEAC Inc., Florence, Italy) following the instructions provided with reagents purchased from Stanbio Laboratories (Texas, USA).

### 2.5. Digestibility Trial

After the feeding trial, the remaining shrimp in each treatment were used for the digestibility test with three replicates of 20 shrimps per tank (240 L). Shrimp were fed their respective diet containing 1% chromic oxide (Cr_2_O_3_, Sigma-Aldrich, USA) four times daily (08 : 00, 11 : 30, 15 : 00, and 18 : 30 h). Rearing water was exchanged 30 minutes after 08.00, 11.30, and 15.00 h feeding to remove leftover feeds and feces on the bottom of tanks. Feces were collected from bottoms of each tank by siphoning three times daily (10 : 30, 14 : 00, and 17 : 30 h) for one week. Feces were not collected after the last feeding at 18.30 h. Collected feces were placed on a filter paper to drain seawater and then frozen at –20°C until analysis. Before freeze-drying, the daily fecal samples from each tank were pooled together. The concentrations of Cr_2_O_3_ in the diets and feces were detected according to Divakaran et al. [42]. Apparent digestibility coefficients (ADC) of protein (ADCp) and dry matter (ADCdm) were calculated according to Cho et al. [43].

### 2.6. Intestinal Histomorphology

Intestine samples were dehydrated using ethanol series and equilibrated in xylene. Samples were coated with paraffin wax to make solid wax blocks. Then, paraffin blocks were sectioned (6 *μ*m thick) with a rotary microtome (Leica RM 2125 RT, Germany), and multiple cross-sections were placed on a glass slide. Slides were dried at 35°C for 24 hours and stained with hematoxylin and eosin. Intestine samples were observed using a microscope Leica DM 750 built with a camera (Leica ICC50E). The morphometric measurements of villi were measured using image analyzing software (Leica Application Suite, version 4.13.0, Switzerland).

### 2.7. Statistical Analysis

Experimental diets were assigned using a completely randomized design. Data of all evaluated criteria were initially checked for normality. When normality assumptions were met, the means of all the parameters were compared by one-way analysis of variance (ANOVA) after arcsine transformation in SPSS version 20.0 (SPSS Inc., Chicago, IL, USA). The results were presented as mean ± standard deviation. When values were found to be significant, a post hoc comparison of means was performed using Tukey's multiple range test with a significance level of 5% (*p* < 0.05). A follow-up trend analysis by orthogonal polynomial contrasts was run to determine whether the effect is linear and/or quadratic. A broken-line regression model was adopted for quantifying the riboflavin requirement based on WG (%) [44].

## 3. Results

**Figure 1:**
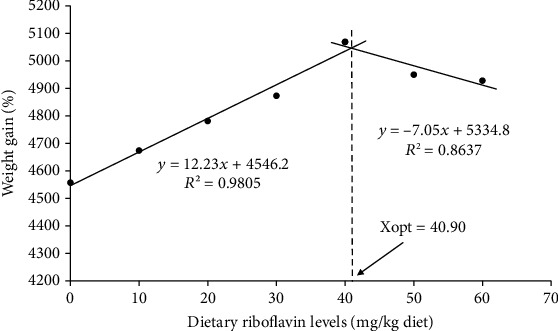
Relationship between weight gain (%) and dietary riboflavin in levels based on broken-line regression analysis where Xopt represents the optimum dietary riboflavin requirement for maximum weight gain (%) of Pacific white shrimp (*Litopenaeus vannamei*).

The growth performance, feed utilization, and survival of *L. vannamei* fed diets were provided in Table 2. The growth performance of shrimp was gradually increased with the increase of riboflavin up to 40 mg/kg diet. R0 group exhibited the lowest growth performance which was significantly lower than all other groups except for R10 group. Final body weight (FBW), WG, and SGR of shrimp fed R40 diet were significantly higher (*p* < 0.05) than those of shrimp fed R0, R10, R20, and R30 diets. However, FBW, WG, and SGR of R50 and R60 groups were comparable with those of shrimp fed R20, R30, and R40 diets. A significantly higher PER value was observed in shrimp fed R40 diet compared to shrimp fed R0 diet. However, FCR and survival were not significantly affected by the increment of dietary riboflavin. FBW, WG, and SGR exhibited significant linear and quadratic trends while FCR and PER exhibited significant quadratic trends to the dietary riboflavin. The optimal dietary riboflavin level was estimated as 40.9 mg/kg diet according to the two-slope broken-line model for growth (Figure 1).

Innate immune responses and antioxidant activities are provided in Table 3. The highest activities of PO, NBT, SOD, and GPx were observed in shrimp fed R40 diet. PO activity was significantly higher in R40 group compared to that of R0 and R10 groups. Lysozyme activity was significantly higher in shrimp fed R30 and R40 diets than in shrimp fed R60 diet. NBT activity was significantly higher in R40 group compared to R0, R10, R20, R50, and R60 groups. SOD activity was also significantly higher in R40 group compared to that of R0 group. GPx activity was significantly increased in shrimp fed R40 diet compared to R0, R10, and R20 groups. Interestingly, R0 group showed the lowest value, which was significantly lower than all other groups. Antiprotease and muscle TAC were not significantly affected by the dietary riboflavin increment. Increased levels of dietary riboflavin linearly enhanced PO activity while lysozyme, NBT, SOD, antiprotease, and muscle TAC displayed significant quadratic trends. Both linear and quadratic trends in GPx activity were significant with the increase of dietary riboflavin.

Whole-body protein, lipid, and ash levels of shrimp are presented in Table 4. There was no significant difference in the whole-body lipid, protein, and ash content among all the dietary groups. Whole-body ash content only showed a linearly decreasing trend with the increase of dietary riboflavin levels.

Hemolymph biochemical parameters are shown in Table 5. Significant differences were not exhibited in hemolymph glucose, triglyceride, protein, and cholesterol levels.

ADCs for dry matter and protein of the diets are presented in Table 6. The ADCdm and ADCp were not significantly affected by the increase of dietary riboflavin.

**Figure 2:**
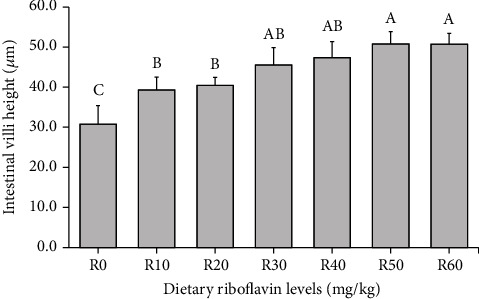
Intestinal villus height of Pacific white shrimp (*Litopenaeus vannamei*) fed the experimental diets for 8 weeks. Bars with different letters are significantly different (*p* < 0.05).

**Figure 3:**
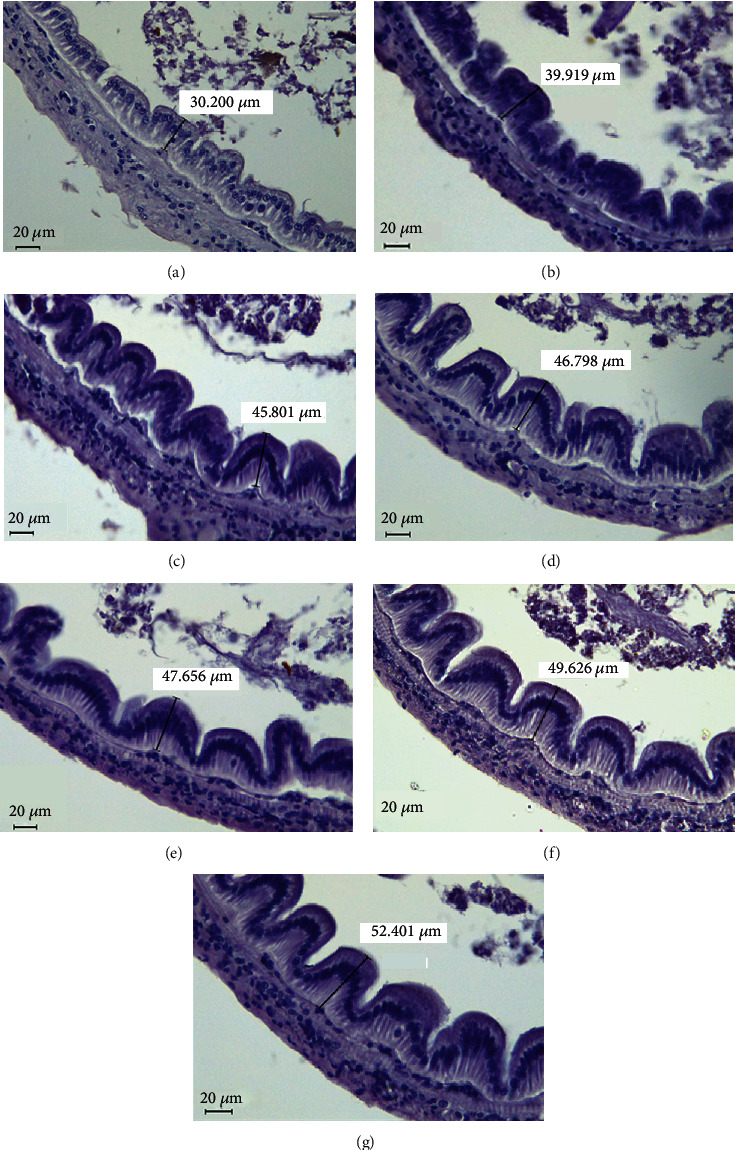
Photomicrographs of transverse HE-stained sections of the intestine of Pacific white shrimp (*Litopenaeus vannamei*) fed seven experimental diets for 8 weeks (villus height; magnification, ×40; scale bar = 20 *μ*m). (a) R0, (b) R10, (c) R20, (d) R30, (e) R40, (f) R50, and (g) R60 (the line segments show the changes in intestinal villus height in experimental groups).

The villus height of shrimp intestine is presented in Figure 2. Villi were significantly longer in shrimp fed R50 and R60 diets compared to shrimp fed R0, R10, and R20 diets. The intestinal villi of shrimp fed R0 diet were significantly shorter than those of shrimp fed other diets. Villi were clearly distinguished in shrimp fed higher levels of riboflavin than in shrimp fed R0 and R10 diets (Figure 3).

## 4. Discussion

The results of this study demonstrated that optimal dietary riboflavin levels could enhance the growth performance and feed utilization efficiency in *L. vannamei.* The optimal riboflavin requirement in this study, which was estimated 40.9 mg/kg, is higher than the requirement for *P. monodon* (22.3 mg/kg) [19] and lower than the requirement for *P. japonicus* (80 mg/kg) [20]. Larval stages of *P. japonicus* require higher vitamin levels than juveniles [45]. Shrimp gut is immature, and they exhibit less vitamin storage capacity in juvenile stage [46]. Accordingly, riboflavin requirements can differ in the growth stages. Riboflavin requirement of shrimp was considerably higher than that of many other aquatic species, such as sunshine bass, 3.45 mg/kg [14]; grass carp, 6.65 mg/kg [13]; blue tilapia, *Oreochromis aureus*, 6 mg/kg [47]; Atlantic salmon, *Salmo salar*, 10-12 mg/kg [48]; and sea cucumber, *Apostichopus japonicus*, 9.73-17.9 mg/kg [49]. Shrimp are robust, bottom-dwelling slow feeders [50]. Dry and sinking pellets are used for shrimp feeding [51]. Derby et al. [52] observed that the first pellets were consumed quickly by shrimp after feeding. Then, they slowly consumed pellets with greater manipulation using their legs and mouthparts. Thus, nutrient leaching from the feeds always occurs while handling the pellets by mouthparts and legs, especially in the case of water-soluble nutrients. Riboflavin and other B vitamins are synthesized by the gut microbiota of animals including fish [53]. However, shrimp has a simple digestive tract compared with fishes, which might lead to a little amount of synthesized riboflavin by their gut microbiota. Energy requirements of animals are usually higher during their rapid development period; however, crustaceans require an extra energy for their molting process [9]. These reasons could explain the difference in riboflavin requirement between fishes and shrimp mentioned above. Coenzyme forms (FAD and FMN) of riboflavin are essential for energy production through the metabolism of major nutrients [1, 54]. Other vitamins, such as pyridoxine, folic acid, and vitamin K, activated by riboflavin were reported to improve the growth of shrimp and prawns [30, 55, 56]. Therefore, these reasons should be part of the reasons for the increased growth of shrimp fed optimum levels of riboflavin in the present study.

PO system is one of the primary components of crustaceans' innate defense system [57]. It is important for the melanization of pathogens [58, 59]. Hemocytes release reactive oxygen species (ROS) during the respiratory burst, which is important to protect the host animals from pathogens by phagocytosis [60]. The present results showed that optimum dietary riboflavin could improve both PO and NBT activities of *L. vannamei*. Lysozyme is a glycolytic enzyme that functions as an antimicrobial agent in the innate immune system and can lead to cell death by cleaving the peptidoglycan component of bacterial cell walls [61]. R30 and R40 groups had significantly higher lysozyme activity, whereas R60 group had the lowest activity. Similarly, high levels of dietary riboflavin reduce the gut lysozyme activity in grass carp [13]. Riboflavin deficiency also decreased lysozyme activity in the intestine and gills of grass carp [12, 13]. Riboflavin reacts with lysozyme under specific conditions, i.e., light-induced binding of riboflavin to lysozyme [62]. Such reactions might be induced by high riboflavin levels resulting in lower lysozyme activity as observed in R60 group in the present study. Future studies are required to evaluate the effect of riboflavin on the lysozyme activity of shrimp. We also suggest that immune-related gene expression should be investigated in future studies to reveal effects of riboflavin on immune responses of shrimp.

Oxidative stress occurs due to higher production of ROS and/or low ability to deactivate ROS [3]. The cellular regulation of ROS is significantly influenced by glutathione (GSH) [63]. The activity of GSH is mediated by the action of GPx [64]. GPx activity in the current study was considerably higher in shrimp fed optimum levels of riboflavin than in the control group. Jiang et al. [11] observed that supplementation of riboflavin could increase GSH concentration in grass carp muscle. GPx activity in the liver, gills, and brain was increased with the riboflavin supplementation in striped catfish [16]. Chen et al. [12] observed that riboflavin supplementation could reduce ROS and increase antisuperoxide anion (ASA) and antihydroxyl radical (AHR) in the gills of grass carp. ASA and AHR can reduce ROS [65]. Riboflavin is a neglected antioxidative vitamin that acts independently or together with the glutathione redox cycle [3, 66]. Riboflavin is likely to improve the antioxidant properties of glutathione which further improves the antioxidant potential in cells by deactivating ROSs [67]. As a result, dietary riboflavin supplementation may be responsible for the increased GPx activity seen in the current study. SOD protects cells from free radical damage [68]. In the present study, optimal dietary riboflavin level (R40 group) significantly increased the hemolymph SOD activity. Riboflavin-deficient diets showed significantly lower SOD levels leading to increased lipid peroxidation in orange-spotted grouper, *Epinephelus coioides* [67], and spotted snakehead [18]. In contrast, relatively high levels of dietary riboflavin tend to reduce both GPx and SOD activities in the hemolymph as observed in R50 and R60 groups in the present study. A similar pattern was seen in grass carp when the dietary riboflavin intake was increased [13]. Riboflavin is an important cofactor for the activation of other vitamins including niacin, pyridoxine, and folic acid [4, 5] which were reported to enhance innate immune responses of shrimps and prawns [28, 30, 55]. Hence, riboflavin might indirectly improve the innate immune response in shrimps. The activities of GPx and SOD are likely to be interfered in R50 and R60 groups due to the independent antioxidant action of riboflavin. TAC level in muscle represents the ability to inhibit lipid peroxidation and its relationship with enzymatic and nonenzymatic antioxidant factors [69]. The different levels of dietary riboflavin in the present study did not affect TAC between the groups, but muscle TAC data showed a significant quadratic trend in response to the riboflavin increase. Therefore, optimum dietary riboflavin level for the maximal antioxidant activity in *L. vannamei* seems to be approximately 40 mg/kg diet.

Histological observations of villi illustrated significant effects of riboflavin on intestinal morphology of *L. vannamei*. Surface area of the gut is expanded by increased villus height. In general, increased villus height indicates improved nutrient absorption [70]. Riboflavin was reported to develop the digestive tract of rats by increasing villus growth and functions because of its function in crypt-sensing mechanism [71]. The crypts are important for gut secretory and absorptive functions of animals [72]. Qin et al. [73] reported that riboflavin regulates renewal and differentiation of intestinal epithelial cells in piglets. They also suggested that riboflavin deficiency can induce intestinal inflammations in piglets. As a result, in the present study, dietary riboflavin intake was found to increase villus height. We assume that epithelial inflammation might be induced in addition to malfunctions of gut crypts of shrimp fed riboflavin-deficient diets because of their lower villus heights. In Jian carp, activities of Na^+^, K^+^, ATPase, and alkaline phosphatase (AKP) were enhanced in intestinal brush border by an optimum dietary riboflavin supplementation [17]. An increase in the activity of Na^+^, K^+^, and ATPase in pig indirectly reflects an improved absorption of amino acids and glucose in the intestine [74]. The activity of AKP was also important to improve nutrient absorption in addition to its ability to develop the gut epithelium [75]. Therefore, sufficient amounts of dietary riboflavin (>40.9 mg/kg) may improve gut morphology and health of *L. vannamei.*

In this study, the effects of riboflavin on the digestibility of dry matter and protein were not significant. Similarly, riboflavin levels had no discernible impact on FCR. The digestibility trial was conducted using the remaining shrimp from each dietary treatment. Therefore, we assume that gut transition time might be lower in riboflavin-deficient groups although the nutrient digestibility was comparable in all the diets. The low gut transition time might have also resulted in low feed consumption leading to lower growth observed in riboflavin-deficient groups in the present study. By contrast, gut transition process might have been accelerated in R50 and R60 groups with increased dietary riboflavin levels. The higher villus growth in the R50 and R60 groups might support this assumption. However, further studies should be conducted to examine the effects of riboflavin levels on gut microbiota in shrimp.

Whole-body ash content was reduced showing a significant linear trend with the riboflavin levels. Whole-body ash level was not significantly different in grass carp [76], Jian carp [17], spotted snakehead [18], and coho salmon (*Oncorhynchus kisutch*) post-smolts [77] fed different levels of riboflavin. However, a decreased whole-body ash content was observed in sea cucumber with increasing dietary riboflavin levels in diets [49]. Effects of riboflavin on body minerals were not well studied in aquatic animals. Riboflavin was reported to improve iron absorption, storage, and mobilization in rats [78] although we observed reduced ash content in whole-body of shrimp. Therefore, further experiments should be conducted to find the effect of riboflavin on the whole-body ash content of shrimp.

Riboflavin affects biochemical parameters of most animals. It is important to maintain and balance cholesterol levels [79]. Wickson and Morgan [80] reported that increased dietary riboflavin levels could reduce blood glucose levels in rats by stimulating glycogenolysis. Kumar [16] observed a similar result in striped catfish. Total protein level in serum was also increased with an increase of riboflavin in stripped catfish [16]. Triglyceride and total cholesterol levels were gradually decreased with the increase of dietary riboflavin levels in Jian carp and coho salmon [17, 77]. However, there was no clear evidence for the riboflavin effect on the biochemical parameters in crustacean hemolymph. Riboflavin levels did not affect the biochemical parameters in the present study. Therefore, biochemical parameters of shrimp might not be affected by riboflavin, or a small amount of riboflavin supplied with the other ingredients in diet might be sufficient in maintaining the balance of biochemical parameters. This should be further investigated in future studies.

## 5. Conclusions

The optimal dietary riboflavin levels can improve the growth performance, feed utilization efficiency, innate immune response, and intestinal villus height of the shrimp. The optimum level of dietary riboflavin seems to be 40.9 mg/kg in the diet for *L. vannamei* based on the growth by a two-slope regression analysis.

## Figures and Tables

**Table 1:** Formulation and proximate composition of the experimental diets for Pacific white shrimp (*Litopenaeus vannamei*) (g/kg, dry matter basis).

Ingredients	Experimental diets						
	R0	R10	R20	R30	R40	R50	R60
Fish meal (sardine)	50.0	50.0	50.0	50.0	50.0	50.0	50.0
Fish meal (tuna)	50.0	50.0	50.0	50.0	50.0	50.0	50.0
Soybean meal	400	400	400	400	400	400	400
Squid liver meal	50.0	50.0	50.0	50.0	50.0	50.0	50.0
Wheat flour	180	180	180	180	180	180	180
Starch	140	140	140	140	140	140	140
Fish oil	50.0	50.0	50.0	50.0	50.0	50.0	50.0
Mineral Mix^1^	20.0	20.0	20.0	20.0	20.0	20.0	20.0
Vitamin Mix^2^	10.0	10.0	10.0	10.0	10.0	10.0	10.0
Cholesterol	2.00	2.00	2.00	2.00	2.00	2.00	2.00
Lecithin	10.0	10.0	10.0	10.0	10.0	10.0	10.0
Monocalcium phosphate	30.0	30.0	30.0	30.0	30.0	30.0	30.0
Riboflavin^3^	0.00	0.01	0.02	0.03	0.04	0.05	0.06
Cellulose	8.00	7.99	7.98	7.97	7.96	7.95	7.94
*Proximate composition* (g/kg)							
Dry matter	927	926	923	928	926	923	926
Crude protein	347	347	343	346	345	344	343
Crude lipid	92.6	85.4	86.1	89.1	86.3	92.1	92.1
Crude ash	91.2	91.2	89.4	89.2	89.2	91.2	92.1
Riboflavin content (mg/kg)	1.41	6.38	17.87	25.26	37.11	46.01	55.11

^1^Mineral mix (g/kg): MgSO_4_.7H_2_O, 80.0; NaH_2_PO_4_.2H_2_O, 370.0; KCl, 130.0; Ferric citrate, 40.0; ZnSO_4_.7H_2_O, 20.0; Ca-lactate, 356.5; CuCl_2_, 0.2; AlCl_3_.6H_2_O, 0.15; Na_2_SeO_3_, 0.01; MnSO_4_.H_2_O, 2.0; CoCl_2_.6H_2_O, 1.0. ^2^Vitamin mix (g/kg): *β*-carotene, 0.57; thiamin hydrochloride, 8.91; niacin 18.0; pantothenate, 18.0; pyridoxine hydrochloride, 7.2; cyanocobalamin, 0.09; ascorbic acid, 100.0; cholecalciferol, 144.0; *α*-tocopherol acetate, 20.0; menadione, 0.9; biotin, 0.9; folic acid, 7.2; inositol, 45.0. ^3^Xi'an Julong Bio-Tech Co., Ltd, Xi'an, China.

**Table 2:** Growth performance, feed utilization, and survival of Pacific white shrimp (*Litopenaeus vannamei*) fed the experimental diets for 8 weeks.

	FBW^1^	WG^2^	SGR (%)^3^	FCR^4^	PER^5^	Survival^6^
R0	7.90 ± 0.22^d^	4557 ± 140^d^	6.62 ± 0.05^d^	1.52 ± 0.08	1.88 ± 0.08^b^	93.0 ± 3.83
R10	8.10 ± 0.12^cd^	4674 ± 76.0^cd^	6.67 ± 0.03^cd^	1.51 ± 0.05	1.90 ± 0.06^ab^	95.00 ± 2.00
R20	8.27 ± 0.12^bc^	4781 ± 73.1^bc^	6.70 ± 0.03^bc^	1.49 ± 0.07	1.97 ± 0.08^ab^	95.0 ± 2.00
R30	8.39 ± 0.17^bc^	4873 ± 69.0^bc^	6.71 ± 0.02^bc^	1.46 ± 0.07	2.05 ± 0.10^ab^	96.0 ± 3.27
R40	8.83 ± 0.10^a^	5069 ± 103^a^	6.80 ± 0.03^a^	1.44 ± 0.04	2.06 ± 0.05^a^	95.0 ± 2.00
R50	8.50 ± 0.10^ab^	4950 ± 14.5^ab^	6.76 ± 0.00^ab^	1.51 ± 0.06	1.92 ± 0.07^ab^	94.0 ± 2.31
R60	8.57 ± 0.16^ab^	4928 ± 109^ab^	6.75 ± 0.04^ab^	1.50 ± 0.06	1.94 ± 0.07^ab^	95.0 ± 3.83
Pr > *F*^∗^						
ANOVA	0.00	0.00	0.00	0.27	0.02	0.84
Linear	0.00	0.00	0.00	0.16	0.12	0.60
Quadratic	0.00	0.01	0.01	0.01	0.03	0.29

Values are mean of quintuplicate groups and presented as mean ± SD. Values with different superscript letters in the same column are significantly different (*p* < 0.05). ^1^Final body weight (g). ^2^Weight gain (%) = [(final body weight–initial body weight)/initial body weight] × 100.^3^Specific growth rate (%) = [(ln final body weight–ln initial body weight)/days] × 100. ^4^Feed conversion ratio = dry feed fed/wet weight gain. ^5^Protein efficiency ratio = wet weight gain/total protein given. ^6^Survival (%) = (final amount of shrimp/initial amount of shrimp) × 100. ^∗^Significance probability is associated with the *F*-statistic.

**Table 3:** Innate immune parameters and antioxidant enzyme activity of hemolymph and muscle total antioxidant capacity of Pacific white shrimp (*Litopenaeus vannamei*) fed the experimental diets for 8 weeks.

	PO^1^	Lysozyme^2^	NBT^3^	SOD^4^	GPx^5^	Antiprotease^6^	TAC^7^
R0	0.26 ± 0.02^b^	13.5 ± 1.89^ab^	3.38 ± 0.01^b^	56.1 ± 0.52^b^	175.2 ± 5.85^d^	10.4 ± 0.59	0.29 ± 0.03
R10	0.27 ± 0.02^b^	17.8 ± 1.87^ab^	3.29 ± 0.11^b^	61.6 ± 3.82^ab^	188.9 ± 5.16^c^	13.0 ± 0.40	0.30 ± 0.05
R20	0.28 ± 0.05^ab^	17.8 ± 2.42^ab^	3.38 ± 0.29^b^	61.7 ± 5.37^ab^	196.2 ± 5.02^bc^	11.0 ± 0.58	0.32 ± 0.04
R30	0.36 ± 0.06^ab^	19.3 ± 2.09^a^	3.73 ± 0.26^ab^	61.6 ± 2.36^ab^	207.8 ± 7.59^ab^	12.5 ± 0.76	0.33 ± 0.02
R40	0.39 ± 0.06^a^	18.9 ± 3.66^a^	3.88 ± 0.17^a^	63.7 ± 2.88^a^	210.2 ± 7.40^a^	12.6 ± 0.40	0.32 ± 0.02
R50	0.35 ± 0.03^ab^	15.8 ± 2.09^ab^	3.30 ± 0.25^b^	63.2 ± 2.41^ab^	204.4 ± 5.59^ab^	10.6 ± 0.74	0.29 ± 0.04
R60	0.34 ± 0.07^ab^	12.3 ± 0.96^b^	3.30 ± 0.24^b^	58.1 ± 1.96^ab^	200.3 ± 5.59^abc^	10.1 ± 2.99	0.32 ± 0.05
Pr > *F*^∗^							
ANOVA	0.01	0.01	0.00	0.02	0.00	0.01	0.38
Linear	0.00	0.36	0.64	0.18	0.00	0.22	0.80
Quadratic	0.06	0.00	0.00	0.00	0.00	0.01	0.03

Values are mean of quintuplicate groups and presented as mean ± SD. Values with different superscript letters in the same column are significantly different (*p* < 0.05). ^1^Phenoloxidase activity (absorbance). ^2^Lysozyme activity (*μ*g/ml). ^3^Nitro blue tetrazolium activity (absorbance). ^4^Superoxide dismutase (% inhibition). ^5^Glutathione peroxidase (mU/mL). ^6^Antiprotesase activity (% inhibition). ^7^Total antioxidant capacity (*μ*mol/mL). ^∗^Significance probability is associated with the *F*-statistic.

**Table 4:** Proximate composition (% wet weight basis) of Pacific white shrimp (*Litopenaeus vannamei*) fed the experimental diets for 8 weeks.

	Protein	Ash	Lipid
R0	18.20 ± 1.98	3.40 ± 0.47	1.46 ± 0.01
R10	18.29 ± 1.14	4.45 ± 0.16	1.39 ± 0.27
R20	18.89 ± 0.73	3.96 ± 0.86	1.34 ± 0.15
R30	18.62 ± 1.53	3.72 ± 0.21	1.39 ± 0.03
R40	19.38 ± 0.62	3.45 ± 0.46	1.14 ± 0.07
R50	19.72 ± 1.52	3.24 ± 0.39	1.52 ± 0.02
R60	18.01 ± 1.80	2.92 ± 0.11	1.51 ± 0.22
Pr > *F*^∗^			
ANOVA	0.40	0.12	0.28
Linear	0.98	0.03	0.66
Quadratic	0.37	0.40	0.09

Values are mean of quintuplicate groups and are presented as mean ± SD. Values with different superscript letters in the same column are significantly different (*p* < 0.05). ^∗^Significance probability is associated with the *F*-statistic.

**Table 5:** Hemolymph biochemical parameters of Pacific white shrimp (*Litopenaeus vannamei*) fed the experimental diets for 8 weeks.

	Glucose^1^	Triglyceride^2^	T. Protein^3^	T. cholesterol^4^
R0	536 ± 3.48	10.7 ± 1.80	3.33 ± 1.19	13.3 ± 1.43
R10	533 ± 7.97	9.32 ± 1.12	3.40 ± 1.01	13.4 ± 1.53
R20	544 ± 7.20	9.50 ± 1.96	3.43 ± 1.39	12.4 ± 1.30
R30	547 ± 6.52	10.9 ± 1.48	3.84 ± 0.93	13.4 ± 1.54
R40	533 ± 6.46	9.90 ± 1.61	3.75 ± 0.71	12.9 ± 1.25
R50	535 ± 10.3	10.1 ± 1.28	3.73 ± 0.78	12.9 ± 2.65
R60	538 ± 12.8	9.54 ± 3.10	3.60 ± 1.47	12.4 ± 1.72
Pr > *F*^∗^				
ANOVA	0.64	0.80	0.93	0.98
Linear	0.89	0.71	0.37	0.56
Quadratic	0.40	0.90	0.50	0.89

Values are mean of quintuplicate groups and presented as mean ± SD. Values with different superscript letters in the same column are significantly different (*p* < 0.05). ^1^Glucose (mg/dL). ^2^Triglyceride (mg/dL). ^3^Total protein (g/dL). ^4^Total cholesterol (mg/dL). ^∗^Significance probability is associated with the *F*-statistic.

**Table 6:** Apparent digestibility coefficients (%, ADC) for dry matter and protein of the experimental diets for Pacific white shrimp (*Litopenaeus vannamei*).

	ADCdm (%)^1^	ADCp (%)^2^
R0	93.1 ± 0.62	86.1 ± 0.24
R10	93.8 ± 0.44	86.9 ± 0.93
R20	94.1 ± 1.02	86.6 ± 2.31
R30	94.0 ± 1.11	87.1 ± 2.39
R40	94.3 ± 0.15	87.9 ± 0.91
R50	94.1 ± 0.43	86.7 ± 0.95
R60	93.6 ± 0.85	86.2 ± 1.84
Pr > *F*^∗^		
ANOVA	0.64	0.85
Linear	0.43	0.78
Quadratic	0.10	0.24

Values are mean of quintuplicate groups and presented as mean ± standard deviation. Values with different superscript letters in the same column are significantly different (*p* < 0.05). ^1^Apparent digestibility coefficient of dry matter (%) = 100 − 100 × (%Cr_2_O_3_ in diet/%Cr_2_O_3_ in feces). ^2^Apparent digestibility coefficient of a protein (%) = 100 − 100 × (%Cr_2_O_3_ in diet/%Cr_2_O_3_ in feces) × (%protein in feces/%protein in diet). ^∗^Significance probability is associated with the *F*-statistic.

## Data Availability

All data generated or analyzed during this study are included in this article.
